# Are children’s judgments of another’s accuracy linked to their metacognitive confidence judgments?

**DOI:** 10.1007/s11409-021-09263-x

**Published:** 2021-03-27

**Authors:** Carolyn Baer, Puja Malik, Darko Odic

**Affiliations:** 1 Department of Psychology, University of British Columbia, 2136 West Mall, Vancouver, BC V6T 1Z4 Canada; 2 Department of Psychology, University of California, Berkeley, 2121 Berkeley Way West, Berkeley, CA 94720 USA

**Keywords:** Confidence, Selective social learning, Signal detection theory, Simulation theory, Individual differences

## Abstract

The world can be a confusing place, which leads to a significant challenge: how do we figure out what is true? To accomplish this, children possess two relevant skills: reasoning about the likelihood of their own accuracy (metacognitive confidence) and reasoning about the likelihood of others’ accuracy (mindreading). Guided by Signal Detection Theory and Simulation Theory, we examine whether these two self- and other-oriented skills are one in the same, relying on a single cognitive process. Specifically, Signal Detection Theory proposes that confidence in a decision is purely derived from the imprecision of that decision, predicting a tight correlation between decision accuracy and confidence. Simulation Theory further proposes that children attribute their own cognitive experience to others when reasoning socially. Together, these theories predict that children’s self and other reasoning should be highly correlated and dependent on decision accuracy. In four studies (*N* = 374), children aged 4–7 completed a confidence reasoning task and selective social learning task each designed to eliminate confounding language and response biases, enabling us to isolate the unique correlation between self and other reasoning. However, in three of the four studies, we did not find that individual differences on the two tasks correlated, nor that decision accuracy explained performance. These findings suggest self and other reasoning are either independent in childhood, or the result of a single process that operates differently for self and others.

**Supplementary Information:**

The online version contains supplementary material available at 10.1007/s11409-021-09263-x.

How do children distinguish truths from falsehoods, like determining that their sibling is lying about the moon being made of cheese? As early as infancy, humans possess several tools to help evaluate truthfulness, including early-emerging core concepts for physical objects and psychological agency (Carey, 2009; Spelke & Kinzler, 2007), and a tendency to trust others (Csibra & Gergely, 2009; Harris, 2012). While these cognitive tools are generally helpful, over-reliance on any single one could lead to mistaken beliefs about the world. For example, if we relied only on our pre-existing concepts, we might never learn counterintuitive truths like the Earth’s sphericity. If we relied only on trusting others’ knowledge, we could fall prey to mischievous lies about cheesy moons. To avoid this, children and adults must consider the reliability of their own knowledge and that of the information provided by others to determine which ideas and concepts to trust and which to disregard.

Accordingly, children have at least two abilities that help them evaluate the reliability of evidence, one focused on the self and one focused on others. First, in part through their broader metacognitive toolbox, children can reason about their own confidence: the graded signal of whether an answer is likely to be true (Flavell, 1979; Pouget et al., 2016). For instance, our sense of confidence might tell us we are likely or unlikely to land a jump, that we might need to double-check an answer on an exam, or that we’re pretty sure the moon is made of rocks. As some evidence for this ability in childhood, preschoolers report higher confidence when they correctly identify a pixelated object or remember seeing an object than when they are incorrect (Hembacher & Ghetti, 2014; Lyons & Ghetti, 2011) and can both prospectively and retrospectively judge their accuracy on simple perceptual discrimination tasks (Baer & Odic, 2019). Twenty-month-old infants similarly respond to uncertainty by asking their caregiver for help (Goupil et al., 2016). These findings suggest that reasoning about confidence, evaluating the reliability of one’s own knowledge, emerges at a young age.

Second, children also detect the reliability of knowledge in *others*. Decades of research in *mindreading* (also known as mentalizing or Theory of Mind) demonstrates that by at least age 3, children detect ignorance in others and use this to predict another’s behavior or choose when to offer help (Liszkowski et al., 2008; Onishi & Baillargeon, 2005; Wimmer & Perner, 1983). Infants and children also make strategic decisions about which people to believe by tracking past accuracy, group membership, confidence displays, and other cues to accuracy (Birch et al., 2010; Koenig et al., 2004; Mills, 2013; Poulin-Dubois & Brosseau-Liard, 2016), which could help children discount statements about cheesy moons from lying siblings. For instance, when only one of two teachers consistently gives the correct labels for objects, preschool children selectively trust the accurate labeller’s future answers and ask her for help instead of the inaccurate labeller (Koenig et al., 2004). Children are therefore also sensitive to the likelihood of others’ knowledge when discerning the truth, rather than being entirely gullible to incoming information.

While extensive work has shown both that children have a metacognitive ability to evaluate their own confidence and are sophisticated in how they evaluate the reliability of others, these two research programs have remained separate, in part because they concern distinct targets: the reliability of self vs. another. However, several theorists have pointed out the potential for overlap between self and other reasoning (Carruthers, 2009; Gopnik, 1993; Proust, 2012). Most notably, reasoning about the self and about others involves thinking about an individual’s knowledge, and specifically the likelihood that the knowledge in question is correct. Accordingly, children could base both self and other evaluations on similar information (e.g., noting for themselves and also for others that long decision times signal answers that are more likely to be wrong; Koriat & Ackerman, 2010b), and could therefore rely on similar processing mechanisms when using this information to form reliability estimates in both cases.

In support, several studies have documented correlations and commonalities between self-focused metacognitive abilities (including reasoning about confidence specifically) and other-focused mindreading abilities (e.g., Gopnik & Astington, 1988; Kuzyk et al., 2020; Lecce et al., 2015; Lockl & Schneider, 2007; Paulus et al., 2014; but see Bernard et al., 2015; van Loon & van Pol, 2019). In one study, three-year-old’s mindreading abilities measured through a battery of false belief tasks predicted their metacognitive knowledge of memory strategies (e.g., spending more time to memorize difficult items) at age 5 (Lockl & Schneider, 2007). Another recent study presented 18-month-old infants with non-verbal self and other reasoning tasks, using a measure of persistence to index confidence (Kuzyk et al., 2020). Infants who poorly monitored their confidence (i.e., persisted on tasks that had no probability of success) were also more likely to learn a new object label from an unreliable teacher (a sign of poor social reasoning; Kuzyk et al., 2020). At the same time, however, some studies find no correlations between reasoning about the self and others. Bernard et al. (2015) found no correlation between preschool children’s metacognition in an opt-out paradigm (where they can choose not to answer when uncertain) and their performance on false belief tasks, a finding replicated in a cross-cultural sample of German and Japanese children (Kim et al., 2020). It thus remains unclear when, if ever, metacognition and mindreading should correlate in childhood and what that means for the way the two abilities operate in the mind.

There are many possible explanations of how reasoning about the self and about others could be linked, and we devote some space in the General Discussion to those not tested here. Here, we test one relatively simple explanation: both judgments about one’s own confidence and about another’s likely accuracy are rooted in children’s understanding of the task that the self and other are doing. Children who understand the task well are better positioned to notice deviations in knowledge and performance in both the self and others, while children who do not understand the task well are not. Importantly, this explanation predicts that there should be correlations in reasoning about expected accuracy when the self and other are doing the same task, but not necessarily when completing different tasks.

This potential mechanistic link between reasoning about the reliability of self and others that is rooted in Signal Detection Theory (SDT) and Simulation Theory. SDT, a highly influential theory in the fields of perception and memory, is designed to explain decision-making under uncertainty (Green & Swets, 1966). The critical observation of SDT is that our internal representations of the world are imprecise, leading to a degree of uncertainty in every decision we make (e.g., what we estimate to be 300 words on a page could plausibly be 237 or 413 or anything within that range; Dehaene, 2011; Green & Swets, 1966). When there is more variability in our decision (e.g., a range of 100–1000 words), we should be less confident about our accuracy; when there is more precision in that decision (e.g., a much narrower range of 299–301 words), we should experience much more confidence. In fact, SDT proposes that our confidence should be *directly* proportional to the degree of variability (Alais & Burr, 2004; Mamassian, 2016). In this way, confidence is much like a standard deviation around a mean, which quantifies the variability or uncertainty around the true mean (fittingly leading to a larger “confidence interval”): when the information we get is imprecise, we should lower our confidence so as not to trust incorrect information. Because SDT provides a computationally simple mechanism for reasoning about confidence, it is an appealing account of how metacognitive confidence evaluations arise (Galvin et al., 2003; Kiani & Shadlen, 2009; Mamassian, 2016; Maniscalco & Lau, 2012; Pleskac & Busemeyer, 2010). Further, it can easily be tested by manipulating the amount of imprecision in a given decision, say by masking an item to be identified or by changing the ratio between two magnitudes to be close (high imprecision) or far (low imprecision).

On its own, SDT makes no explicit prediction about whether reasoning about confidence in the self vs. others is a single process or not; it simply states that if you have an imprecise internal representation, your expected accuracy (confidence) can be estimated from the amount of internal variability. However, a popular theory within the mindreading literature, *Simulation Theory* (Goldman, 2006; Jost et al., 1998; Meltzoff, 2007; Nickerson, 1999), could accommodate this link. Under this view, interpreting others’ mental states occurs through a simulation process, metaphorically putting oneself in another’s shoes, and attributing the experienced mental states to that other. When a child is reasoning about another’s likelihood of accuracy, then, this simulation would involve reasoning metacognitively about their *own* likelihood of accuracy but attributing that likelihood to another person.

As evidence for Simulation Theory, first-hand experience seems to enable children to detect mental states in others when they otherwise wouldn’t, suggesting that reasoning about the self is intricately linked to and predictive of reasoning about others. For instance, 3-month-old infants who gain experience grasping a desired object by wearing sticky mittens (something otherwise difficult for 3-month-olds) can then detect the goals of a grasping hand (Sommerville et al., 2005), even though 3-month-olds without this experience do not infer these goals (Woodward, 1998). Within the realm of confidence judgments specifically, when adults and children make judgments of learning (a type of confidence judgment based on one’s expectations about how successful future performance on an item will be), they are more accurate in predicting others’ learning if they have first predicted their own performance (Koriat & Ackerman, 2010b; Paulus et al., 2014). In fact, reasoning about others can go awry if the metacognitive process itself is misled: adults who have learned and forgotten trivia items are more likely to attribute knowledge of those items to their peers, but not knowledge of items they were never taught (Birch et al., 2017). These findings strongly suggest that our evaluations of others – attributing goals, judging others’ learning, and estimating the prevalence of knowledge –are highly influenced by reasoning about the self.

In combination, then, SDT and Simulation Theory together could explain correlations between own and other reasoning by arguing that both abilities rely on the same key process of quantifying the imprecision of a decision. That imprecision can be attributed to the self as feelings of confidence or to another as an assessment of likely accuracy. For example, if we estimate that there are between 200 and 300 words on this page, we could – using principles of SDT – judge our own confidence of the decision that there are “1000 words” as very low in probability. Similarly, if we hear somebody else estimating that there are “1000 words” on this page, we could likewise use the same process to estimate our confidence in *their* decision, judging them to be an unreliable teacher for future word-estimation decisions. Therefore, any time a child and the person they are observing have access to the same information (e.g., are looking at the same page of words), we should expect to find strong correlations in their estimates of self and other accuracy. This is often true in the studies reporting correlations – children evaluate whether they know what an object really is and then evaluate another’s perspective about the same object (Gopnik & Astington, 1988), or they evaluate whether they or another know the answer to one target memory pair (Paulus et al., 2014).

Besides making identical judgements when given identical information, the combination of Simulation Theory and SDT also makes a second testable prediction. Since SDT proposes that confidence is a direct computation of the imprecision of a decision and Simulation Theory proposes that evaluating another’s likely accuracy is the same as reasoning about your own, the combination of these accounts predicts that individual differences in the imprecision of decisions should be the core source of variability for both self and other judgments. Put differently, if we can directly measure a subject’s decision imprecision and statistically control for it, any correlations between self and other reasoning should disappear.

Therefore, in the studies reported here, we set out to test these two predictions: (1) whether there are strong correlations between detecting accuracy in the self and others when given access to the same task (and not when using an unrelated task), and (2) whether these correlations are eliminated when controlling for individual differences in the imprecision of decisions in that task. We chose to use a task tapping into children’s perception of area, an early-developing ability used previously in metacognitive tasks (Baer & Odic, 2020a; Baer et al., 2018; Salles et al., 2016). This task allows us to experimentally manipulate the imprecision of the decision by making the shapes close in size (harder, more imprecision) or disparate (easier, less imprecision), something much harder to achieve in the memory tasks used in past work. We correlated how well children computed the imprecision in simple area discrimination decisions (i.e., how sure am I that I know which of two shapes is larger) compared to their judgements of how well *others* do it (i.e., which of two agents did better on a shape drawing competition).^1^

In designing this study, we also needed to ensure that any correlations found are not the result of other processes in common to the self and other judgments. For example, many mindreading and metacognition tasks depend on the use of mental state verbs (e.g., “know” or “think”). While the shared language signals that judgments of knowledge in both the self and others contribute to a shared concept of ‘knowledge’, relying on this language may artificially link the processes leading to such judgments, which may themselves be distinct. As one solution, some recent studies have turned to measures of *procedural metacognition* (or reasoning about confidence without requiring mentalistic language). For example, the study by Kuzyk and colleagues (2020) described earlier uses a measure of persistence rather than explicit report, thereby avoiding the confound of developing mental state language that was present in past work. Therefore, following this work, we also chose paradigms that measure children’s self and other reasoning in a way that avoids mentalistic language.

A second major problem is the influence of response biases. Within the metacognition literature, it is well documented that children tend to report higher confidence in their knowledge and abilities than is warranted (e.g., Destan & Roebers, 2015; Hagá & Olson, 2017; Taylor et al., 1994; van Loon et al., 2017), possibly due to overoptimistic beliefs about the self (Lockhart et al., 2017). Similarly, when reasoning about others’ knowledge, children will imitate seemingly irrelevant actions even though they seem to understand the irrelevance (D. E. Lyons et al., 2007; Meltzoff, 1988) and trust adults who blatantly lie to them even when children know the truth (Jaswal, 2010). Given these patterns, which co-occur in childhood (Hagá & Olson, 2017), there is a chance that some of the reported correlations between self and other evaluations might stem from common response biases. For instance, in Kuzyk et al. (2020), some infants might be naturally inclined to seek out as much information as possible when faced with uncertainty both by persisting longer or by trusting adults, leading to correlations that are driven entirely by children’s information-seeking biases and not their metacognitive abilities, per se. Other response biases, like a desire to please adults or to be optimistic, could similarly affect both self and other judgments and lead to correlations even if the two have completely unrelated cognitive processes.

In the current work, we test for correlations between self and other reasoning about accuracy using tasks that eliminate overconfident response biases and mental state language while maintaining the critical commonality deemed necessary under the SDT/Simulation Theory account: task-specific decision imprecision. First, we adopt a relative choice paradigm for both the self and other tasks, rather than relying on absolute judgments like declarations of knowledge or decisions to trust or not trust a teacher. In a relative task, children are asked to indicate which of two options best fits a given criteria (in this case, which is more likely to be true). Notice that in doing so, we remove a child’s ability to respond overconfidently because they must pick one of two answers they cannot simply say ‘yes’ to everything. Instead, they must reason about which of the two options is *the most* likely to be true. In the self reasoning task, this involves selecting which of two questions children feel most sure of answering correctly (e.g., Baer & Odic, 2019; Butterfield et al., 1988). In the other reasoning task, this involves selecting which of two teachers children feel is more reliable (called a selective social learning task; e.g., Birch et al., 2008; Einav & Robinson, 2010; Koenig et al., 2004). By experimentally eliminating these response biases, we can ensure that any correlations between the self and other judgments do not stem from shared response biases.

Then, to limit the use of mental state language, we ask children to make strategic judgments that rely on assessments of knowledge (see Crivello et al., 2018; Hembacher & Ghetti, 2014). For example, instead of asking children to report which teacher knows more, we ask children to make a strategic choice to ask one teacher for help (Einav & Robinson, 2010; Koenig et al., 2004). And, instead of asking children to report whether they know an answer or not, we ask them to strategically answer the question they feel most sure about (Baer & Odic, 2019). These changes help reduce the potential influence of shared linguistic concepts inducing correlations between the two tasks.

The prediction of the SDT/Simulation account is that self and other reasoning on these two tasks should correlate despite the removal of these third variable explanations, provided one critical condition is true. The SDT account requires that they will correlate if and only if they are both computed from the *same* decision imprecision, which is thought to be dimension-specific (Baer et al., 2018; Vo et al., 2014). That is, children’s confidence in their own or another’s estimate of the number of words on a page should be related, but both should be entirely unrelated to their confidence in their own or another’s estimate of the emotional expression on a face. We therefore adapted both paradigms to use a single target decision. Specifically, children were asked to reason about the relative sizes of shapes, tapping into a system of representing area that is well-developed in childhood but still subject to individual differences (Brannon et al., 2006; Odic, 2018). In fact, these individual differences in area representation are thought to be the direct result of representational imprecision – the more imprecise a child’s perceptual representation of area, the harder it will be for that child to tell apart two sizes (Brannon et al., 2006; Odic, 2018; Odic et al., 2013). Therefore, to test the second prediction that any correlation between the self and other tasks should be eliminated when controlling for imprecision, we can use children’s accuracy on these area discriminations to capture the imprecision in their sense of area.

Together, by using two paradigms that eliminate response biases and mental state language as potential third variables, and by using a single type of representation in both paradigms, we can test whether SDT and Simulation Theory together can explain how children reason about the likely accuracy of their own and other’s knowledge. In four studies, we test whether self and other reasoning is correlated when representations are shared, and uncorrelated when representations are distinct (the first prediction). Then, in Experiments 3 and 4, we use two techniques to test whether shared representations entirely explain any correlations (the second prediction). To anticipate our findings, we surprisingly failed to detect a consistent correlation between self and other reasoning in the studies – a basic requirement of the SDT/Simulation account. We therefore also conducted a mega-analysis of all four studies to clarify the results.

## Experiment 1

### Methods

#### Participants

A total of 80 children participated in the study (44 girls), meeting the planned sample size of 80 children (20 per age group, set arbitrarily a priori, see Simmons et al., 2011). We focused on children between 4 and 8 years (*M* = 5;11 [years; months], range = 4;0—7;10) to overlap the age ranges of studies using similar paradigms (Baer & Odic, 2019; Einav & Robinson, 2010). Both these studies show development in these skills over this age range, which additionally helps us find individual differences that should correlate between the two tasks according to the SDT/Simulation theory account. Five additional children were tested but not included in the sample because they did not complete the study. Children were tested individually in a quiet area of their schools and daycares. All children spoke enough English to carry on a short conversation, and were predominantly White or South-East Asian and middle-class, as is representative of the Greater Vancouver Area in British Columbia, Canada.

#### Materials and procedures

To test whether children’s evaluations of self and other accuracy correlate when using the same representational variability, we adapted two existing paradigms: the selective social learning paradigm (reasoning about others) and the relative confidence task (reasoning about the self). Children completed the two tasks in a fixed order, with the selective social learning task first and the confidence discrimination task second. Both tasks were presented on a laptop.

##### Selective social learning task

To assess individual differences in children’s reasoning about others’ knowledge, we used a selective social learning paradigm modified to rely on area representations. In the selective social learning paradigm, two informants are shown to differ on a critical trait (e.g., past accuracy, displayed confidence, social group, etc.) and children are asked to make a series of social judgments to indicate which informant they find most reliable. If children consistently choose one informant over the other, we can reason that children notice and care about the critical trait (e.g., Birch et al., 2010; Koenig et al., 2004). Here, we based our task on a variation by Einav and Robinson (2010) in which one informant is consistently more accurate than the other during a short ‘history’ phase. Importantly, neither informant is truly accurate – both informants provide incorrect answers that differ only in the magnitude of their error. For example, in Einav and Robinson’s study, informants said that either 6 or 10 dots were on a card when there were actually 5. Because children cannot evaluate accuracy here by identifying the correct or incorrect item (as both are incorrect), children must rely on their evaluations of the magnitude of each informant’s error, i.e., the relative likelihood of their accuracy.

In our modified version using area representations, children saw photos of a pair of “contestants in a drawing contest” and were asked to help the experimenter “choose the winner”. At the beginning of the study, the experimenter told children that the contestants had to copy a shape perfectly, and that it was particularly important for the shape to be the same size (children were asked to repeat this rule to ensure understanding). We felt that using differences in size, rather than numerosity as Einav & Robinson did, would make the task more accessible to children who were still learning number words (Le Corre & Carey, 2007).

**Fig. 1:**
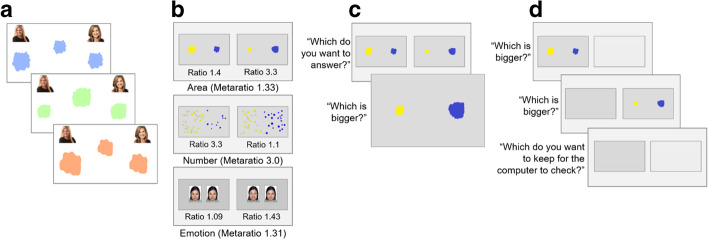
Selective social learning task and confidence task stimuli. Panel A depicts a sample trial in the selective social learning task. Children saw three examples of two informants’ drawings beside a target object. The right contestant is the ‘closer’ contestant in this example. Children then made Winner, Ask, and Endorse judgments about this pair of contestants. Panel B depicts sample stimuli from the confidence task in Experiment 1. Only the area trials were used in Experiments 2–4. Panel C depicts the confidence paradigm used in Experiments 1, 2, and 4. Children first selected which of two questions they wanted to answer, then answered only that question. Panel D depicts the confidence paradigm used in Experiment 3. Children first answered each question, then selected which answer they felt most confident about

To obtain coarse individual differences, children completed 4 trials, each with three judgments of informant accuracy. In each trial, children were introduced to a new pair of White female “contestants”. Because the SDT/Simulation account proposes that only representational imprecision is used to compute confidence, we used only photographs of the contestants rather than videos or live actors to remove other potential cues to confidence like reaction time or movement cues (see Kominsky et al., 2016 for a similar method). Children then saw three drawings from each contestant which critically differed only in size. Each example started with a target shape in its true size in the center of the screen, followed by the “copies” made by each contestant underneath their respective photos (see Fig. 1). Across the three examples within each trial, one contestant consistently produced shapes that were relatively closer in size to the target (either larger or smaller than the target by a ratio of 1.2; e.g., 120% or 83% of the original size), while the other contestant produced relatively further-sized shapes (a ratio of 2.0 – 200% or 50% – over or under in the same direction as the ‘closer’ contestant). Shapes varied in whether they were too large or too small between examples to avoid children learning a rule that the smallest/largest shape was always the winner. The left/right positioning of the closer-sized shape was counterbalanced across the 4 trials, and the identities of the ‘winning’ contestants were counterbalanced between participants.

Following the three examples in each trial, children answered three test questions based on classic selective social learning measures (e.g., Birch et al., 2008; Einav & Robinson, 2010; Koenig et al., 2004). First, the experimenter asked children to choose the “Winner” of the contest (i.e., who drew their shapes closer in size to the targets), providing a direct assessment of whether children detected the difference in relative accuracy. Children were then told they could ask one of the two contestants for assistance on a drawing contest to be held in class, allowing us to see if their assessments of accuracy carry over to their judgments of worthy teachers (an “Ask” judgment). Finally, the experimenter pretended as though she was showing another example drawn by the contestants, but the target shape didn’t show up because of a “computer glitch.” Instead, children saw shapes of the same size drawn by the two contestants and were asked to indicate which shape was probably more like the target [that didn’t show up]. Thus, much like “Endorse” trials in other studies (e.g., Koenig & Harris, 2005), we expected children to rely on their previous judgments of competency to make their selection, given the absence of an objective answer.

##### Confidence task

To assess individual differences in children’s sensitivity to confidence, we administered the Relative Confidence Task from Baer et al. (2018). In each trial, children had to make a simple choice, like whether a yellow or blue shape is larger (see Fig. 1 and Odic, 2018). Critically, and following the principles of SDT, this task manipulates the degree of confidence participants should feel in the choices by varying specific properties of the stimuli. For instance, in the case of area judgments, when the ratio of pixels in the blue and yellow shapes is large like ratio 3.3 (e.g., 119,130 yellow pixels and 36,100 blue pixels), participants should experience higher confidence than in smaller ratios like 1.05 (Baer et al., 2018).

To assess sensitivity to these differences in confidence, children saw screenshots of area comparisons in pairs on the screen prior to answering and selected which of the two screenshots they “wanted to answer,” appealing to a desire to answer correctly. Effectively, then, we were asking children to compare the sizes of shapes in two questions, then compare their confidence in each of those size comparisons. Children then answered only the selected question. Screenshots were paired to make three “metaratios”: differences in difficulty between the two screenshots. For example, one trial with a ratio of 3.3 on the left and a ratio of 1.1 on the right yields a metaratio of 3.0 (3.3 / 1.1). By varying the difference in difficulty, we can identify children who can tell apart only large differences between their confidence (e.g., the difference between “very sure” and “not sure”) versus children who can tell apart even small differences in their confidence (e.g., between “very sure” and “somewhat sure”), yielding a measure of individual differences.

The confidence task included 3 independent perceptual dimensions: area, number, and emotion (detailed below; Baer et al., 2018; Odic, 2018; Vo et al., 2014). By extracting confidence judgments from children on each of the three dimensions, we can test the first prediction of the SDT/Simulation account that self and other reasoning should *only* correlate when representations are shared. That is, we would expect area confidence reasoning to correlate with our area-focused Selective Social Learning Task, but not number confidence or emotion confidence.^2^

On each Area question, children selected whether a yellow or blue shape was bigger (see Fig. 1 and Odic, 2018). Expected confidence in these choices was manipulated through the ratio of pixels in the blue and yellow shapes (e.g., a ratio of 3.3 yellow pixels for every blue pixel for high confidence or a ratio of 1.05 for low confidence; Baer et al., 2018). There were 5 ratios in total for this task (3.3, 2.1, 1.4, 1.1, and 1.05), which were then paired into three metaratios (3.0, 2.0, and 1.33). Metaratio pairs were created such that the largest shape was not always in the high confidence pairing to prevent children from using a heuristic like ‘choose the largest of the 4 visible shapes.’ On each Number question, children selected whether a set of yellow or blue dots was more numerous (see Fig. 1 and Halberda et al., 2008). Here, expected confidence was manipulated through the ratio of dots in the blue and yellow sets using the same ratios as the area questions (3.3, e.g., 33 yellow dots and 10 blue dots, 2.1, 1.4, 1.1, and 1.05). Metaratio pairs, like the area trials, were 3.0, 2.0, and 1.33. In each Emotion question, children selected which of two expressions was happier (see Fig. 1 and Baer et al., 2018; Vo et al., 2014). The expressions, taken directly from Baer et al. (2018), were created by blending a happy and angry expression by one of four female models (two Caucasian and two East Asian). The blended expressions ranged from 100% happy (i.e., 0% angry), through 53.3% happy (i.e., 46.7% angry). Expected confidence was manipulated by varying the ratio of the happy/angry weights (e.g., 93.3% happy vs. 60% happy, a ratio of 1.56 for a high confidence trial, 73.3% happy vs. 66.7% happy, a ratio of 1.1 for a low confidence trial). This resulted in 5 different binned ratios (1.09, 1.2, 1.31, 1.43, and 1.57), paired to make metaratios of 1.44, 1.31, and 1.1. See https://osf.io/dtzpq/ for exact stimuli.

In past work with this paradigm, approximately 90% of children strategically choose the easier of the two images, relying on a subjective sense of their own confidence in being able to correctly answer the question (Baer & Odic, 2019; Baer et al., 2018). The remaining 10% of children strategically choose the *harder* of the two images, often citing a desire to challenge themselves. While the response produced by these children is qualitatively different than expected, the underlying ability to detect differences in confidence that we are interested in (i.e., to identify which trial feels ‘easy’ and which feels ‘hard’) remains identical to children who chose the easier option. In fact, given that our analyses rely on correlations, these children could artificially induce correlations where none otherwise exist. However, these children can objectively be identified using a psychophysical model that expects children to be increasingly likely to select the easier question as the difference in difficulty increases. If children become *less* likely to select the easier question (i.e., they consistently pick the harder question), their data will only fit an inverted model, allowing us to identify these children and invert their data (see Baer et al., 2018 for a description of the model).

The Confidence Task consisted of 45 trials in total (five at each of three metaratios in the three dimensions), with 4 warm-up trials of the Area, Number and Emotion tasks alone (i.e., with no preceding confidence choice). To keep children engaged, the three dimensions were randomly intermixed and children received pre-recorded feedback from the computer (“Yeah, that’s right!”, “Oh, that’s not right.”) when they answered the Area, Number, and Emotion questions (e.g., which shape was larger). They received no feedback about their confidence choices (see Baer & Odic, 2019 for evidence that this feedback does not affect performance relative to neutral affirmations).

### Results

#### Selective social learning task

First, we examined whether children’s performance on the Selective Social Learning task replicated typical patterns in each of the three response types: Winner, Ask, and Endorse. At ages 6 and 7, children reliably chose the closer contestant as the Winner, though children at age 4 did not (see Table 1 for means and tests against chance of 50%). Performance correlated with age, *r*(78) = 0.48, *p* = 0.001, indicating that older children were better at detecting and attributing the differences in sizes to the contestants. Similarly, 7-year-olds reliably Asked the closer contestant for help, while children aged 4 through 6 did not (see Table 1). There was a significant correlation with age, *r*(78) = 0.24, *p* = 0.036, suggesting that older children were more likely to use their judgments of error magnitude to inform their help-seeking.

**Table 1:** Tests against chance at each age group in experiments 1 and 2

	Experiment 1					Experiment 2				
	Mean (%)	SD	*t*(79)	*p*	*d*	Mean (%)	SD	*t*(80)	*p*	*d*
Winner										
4	56.25	21.27	1.31	0.204	0.29	40.00	26.16	-1.71	0.104	0.38
5	57.14	23.90	1.37	0.186	0.30	64.29	26.89	2.43	0.024	0.53
6	77.63	24.85	4.85	< 0.001	1.11	86.25	28.65	5.66	< 0.001	1.27
7	85.00	23.51	6.66	< 0.001	1.49	86.25	23.61	6.87	< 0.001	1.54
Ask										
4	51.25	23.61	0.24	0.815	0.05	47.50	22.80	-0.49	0.629	0.11
5	59.52	26.78	1.63	0.119	0.36	67.86	23.90	3.42	0.003	0.75
6	60.53	34.68	1.32	0.202	0.30	63.75	33.91	1.81	0.086	0.41
7	72.50	27.98	3.60	0.002	0.80	80.00	28.79	4.66	< 0.001	1.04
Endorse										
4	50.00	25.65	0.00	1.00	0.00	41.25	24.70	-1.58	0.130	0.35
5	57.14	27.55	1.19	0.249	0.26	46.43	31.90	-0.51	0.614	0.11
6	52.63	18.44	0.62	0.542	0.14	52.50	35.26	0.32	0.755	0.07
7	62.50	34.89	1.60	0.126	0.36	48.75	32.92	-0.17	0.867	0.04
Area Confidence										
4	63.33	14.43	4.13	0.001	0.92	65.00	12.40	5.41	< 0.001	1.21
5	67.94	8.85	9.29	< 0.001	2.03	68.57	15.94	5.34	< 0.001	1.17
6	69.12	14.09	5.92	< 0.001	1.36	66.67	14.18	5.26	< 0.001	1.18
7	77.00	13.59	8.89	< 0.001	1.99	69.33	14.73	5.87	< 0.001	1.31
Number Confidence										
4	61.33	10.28	4.93	< 0.001	1.10					
5	62.22	11.42	4.91	< 0.001	1.07					
6	65.26	14.33	4.64	< 0.001	1.07					
7	67.33	12.96	5.98	< 0.001	1.34					
Emotion Confidence										
4	60.00	11.03	4.05	0.001	0.91					
5	57.78	11.61	3.07	0.006	0.67					
6	62.81	13.21	4.23	0.001	0.97					
7	61.00	11.70	4.20	< 0.001	0.94					

decisions. However, we found a different pattern of results in children’s Endorsement of one contestant over the other when lacking an objective reference: all age groups chose at chance rates, with no difference between age groups, *r*(78) = 0.18, *p* = 0.114. This was unexpected given that a recent meta-analysis conducted on selective social learning tasks found strong evidence that children endorse informants they believe to be more accurate (Tong et al., 2020).^3^ See the Supplemental Material for additional correlations between measures. Given this result, we report all correlations between this task and our confidence measure separately for each question.

#### Confidence task

In this task, we expected children to select the easier of the two screenshots if they could tell them apart using their subjective confidence. Accordingly, we found that children at all age groups selected the easier trial more than 50% of the time in all three dimensions (see Table 1 for means and tests against chance). For these analyses, children who responded consistently with the harder option (4 in the Area trials: one 4-year-old, one 6-year-old, two 7-year-olds, 18 in the Number trials: seven 4′s, five 5′s, two 6′s, and four 7′s, and 17 in the Emotion trials: five 4′s, seven 5′s, one 6, and four 7′s, detected by the psychophysical model) have been inverted to match the response pattern of the rest of the sample. However, with their original data, this test against chance remains significantly above chance in 4, 5, and 6-year-olds. See the Supplemental Material for additional analyses of this task.

#### Correlations of individual differences

Given that both tasks demonstrated as expected that children were sensitive to relative differences in their own (Confidence Task) or another’s relative accuracy (Selective Social Learning Task, except the Endorse trials), we next looked for correlations of individual differences between the two tasks. As shown in Table 2, we surprisingly found no correlations between the Winner, Ask, or Endorse choices and any of the three dimensions for the confidence task when controlling for age.

**Table 2:** Correlations controlling for age in experiments 1 and 2

	Area Confidence	Number Confidence	Emotion Confidence
Experiment 1			
Winner	-0.08	0.00	-0.05
Ask	-0.05	-0.14	0.06
Endorse	0.02	-0.16	0.05
Experiment 2			
Winner	0.29*		
Ask	0.36**		
Endorse	-0.02		

* denotes p < 0.05, ** denotes p < 0.01, *** denotes p < 0.001

### Discussion

Contrary to our predictions, we found no correlations between the self and other tasks. Although this was expected for the individual differences in emotion and number confidence (as the Selective Social Learning task only tested area and the SDT account predicts that correlations should only occur when the task is the same for both self and other), it was contrary to our expectation for the area Confidence task.

Nevertheless, we replicated two findings in the existing literature on self and other evaluations, signalling that our self and other tasks worked as intended. First, we replicated the key findings of Einav and Robinson’s (2010) magnitude of error selective social learning task, in which children relied on informants who provided relatively more accurate answers. This occurred despite three changes to the paradigm (the use of photographs instead of videos, judgments about area rather than numerosity, and without the use of a number line to track estimates), but did not replicate in 4-year-olds, in children under age 7 in the Ask trials, or in the Endorse trials. We also found that children as young as 4 years could reason about their relative states of confidence for Area, Number, and Emotion judgments, younger than in past reports (Baer & Odic, 2019; Baer et al., 2018), though we did not see an influence of the difference in trial difficulties or significant improvements with age.

One possible explanation for the failed replication is that the length of the confidence task with all three dimensions fatigued children, making it difficult to capture reliable individual variability. Therefore, in Experiment 2, we conducted a replication with only the area trials of the confidence task to reduce possible fatigue effects.

## Experiment 2

### Methods

#### Participants

Eighty-one children participated in the study (*M* = 5;11, range = 4;0—7;10, 50 girls) in the same manner and geographical location as Experiment 1. None of the children had participated in Experiment 1.

#### Materials and procedures

##### Selective social learning task

We used the same ‘drawing contest’ task from Experiment 1, but with one small change: we made the two shapes in the Endorse trials different sizes to make the contrasting answers more noticeable.

##### Confidence task

We used only the 15 Area trials from the confidence task in Experiment 1 to reduce fatigue and potential task-switching effects.

### Results

#### Selective social learning task

Children aged 5–7 reliably chose the closer contestant as the Winner, though children at age 4 did not (see Table 1 for means and tests against chance of 50%), and choice correlated with age, *r*(79) = 0.56, *p* < 0.001. Similarly, children aged 5 and 7 reliably Asked the closer contestant for help, while children aged 4 and 6 did not (see Table 1). There was still a significant correlation with age, *r*(79) = 0.38, *p* = 0.001. Once again, no age group Endorsed the closer contestant above chance rates (see Table 1), and there was no correlation with age, *r*(79) = 0.16, *p* = 0.153. Additional correlations are reported in the Supplemental Material.

#### Confidence task

Children at all age groups selected the easier trial more than 50% of the time (see Table 1 for means and tests against chance). Eleven children consistently selected the harder task (one 4-year-old, three 5-year-olds, three 6-year-olds, and four 7-year-olds), and their data has been inverted. Additional analyses are reported in the Supplemental Material.

#### Correlations of individual differences

In contrast to Experiment 1, and as predicted by the SDT/Simulation account, the Confidence Task correlated with both the Winner and Ask choices, but not with Endorse choices (see Table 2, all correlations controlling for age).

### Discussion

These results provide preliminary evidence for the SDT/Simulation account, with a correlation between the self and other tasks that held when controlling for age. The pattern of findings in the confidence task was largely the same, potentially suggesting that fatigue alone is insufficient to explain the lack of correlation in Experiment 1. However, 5-year-olds in this study chose to ask the closer informant for help, whereas only 7-year-olds did so in Experiment 1. It may therefore be possible that children in this sample, for whatever reason, encoded the difference in accuracy more deeply.

At the same time, these correlations alone only support the first of the two predictions of the SDT/Simulation account. The assumption of the SDT/Simulation account is that this correlation is driven by a common system of representing confidence based on variability in area representations (in this case). While we experimentally eliminated common response biases and the necessity of common mentalistic language as potential third variables, this study does not yet provide evidence that the common area representations are the sole connector between self and other judgments. To examine this directly in Experiment 3, we collected a measure of area discrimination performance as part of the Confidence Task by relying on a retrospective confidence judgement: children first answered two area discrimination decisions (i.e., which of two shapes is larger), and then afterwards decided which of the preceding two trials they were more confident on. By having a measure of both their area perception *accuracy* and their metacognitive *precision*, we can test the prediction that self and other reliability judgements are no longer correlated when controlling for area accuracy.

## Experiment 3

### Methods

#### Participants

Eighty-one children participated in the study (*M* = 6;0, range = 4;0—8;0, 39 girls), in the same manner and geographical location as Experiments 1 and 2. Two additional children were tested but not included in the sample because they did not complete the study. None of the children had participated in the previous experiments. In addition to the two tasks described, we asked parents to complete a short vocabulary assessment online in the two weeks following participation in the study (the Developmental Vocabulary Assessment for Parents, or DVAP; Libertus et al., 2015). We had hoped to use this measure as a coarse approximation for general intelligence, but we had low rates of completion (26 of 81 participants). These data are reported in the Supplemental Material.

#### Materials and procedures

##### Selective social learning task

We used the same ‘drawing contest’ task, but with two small changes. First, to increase the variability of individual differences and potentially make the task possible for 4-year-olds, we modified the degree of error in two of the four trials. As before, two trials featured errors at a ratio of 1.2 (e.g., 120% or 83% of the original size) against 2.0 (200% or 50%), while the other two trials featured a ratio of 1.2 against 3.0 (300% or 33%). Second, we made the two shapes in the Endorse trials exactly the same size to reduce the likelihood that children would rely on alternative heuristics like ‘choose the largest shape’.

##### Confidence task

We modified the Confidence Task so that rather than making prospective judgments about their success, children evaluated their confidence retrospectively (Baer & Odic, 2019, 2020a). Like before, children saw four warm-up trials of the ‘blobs game’ (area discriminations) before being introduced to the confidence portion of the task. The experimenter told children that they would need to get a lot of questions correct in order to win the game, but that the child could choose between pairs of questions and keep the answer they were “more sure” they got right. On each trial, children answered one question on the left side of the screen, then one question on the right side of the screen, and then made a choice about which one they were most sure they got correct. Questions were never visible on the screen at the same time. Area discrimination questions ranged from a difficult ratio of 1.03 to and easy ratio of 3.3 and were paired to form 20 confidence trials at metaratios of 1.1, 1.33, 2.0, and 3.0. Children did not receive feedback in any part of the task, as feedback immediately after their area answers (but before their confidence choice) would have eliminated the need for them to reason metacognitively.

Because all children had to answer the same 40 Area questions, we used their accuracy on these questions as a measure of the imprecision in their area representations. As outlined in the Introduction, the variability that is thought to index confidence under SDT models is the very same variability that makes it difficult to compare two representations together. If a child possesses a very precise sense of area, they might only have difficulty comparing very similar areas to one another. However, if a child possesses an imprecise sense of area, they will be unable to compare even areas that are dissimilar and easy for other children. Because our Area questions all involve comparing two areas, we can thus infer that children whose accuracy is low on the Area questions possess less precise representations than children with high accuracy.

### Results

#### Selective social learning task

Children aged 5 and 7 chose the informant with lesser errors as the winner more often than chance of 50% (see Table 3 for means and tests against chance), and choice correlated with age, *r*(79) = 0.35, *p* = 0.001. Six-year-olds similarly chose the informant with lesser errors, though this did not reach traditional levels of significance (see Table 3). As in Experiment 1, only 7-year-old children chose to Ask this contestant for help significantly more than chance (see Table 3), behavior which also correlated with age, *r*(79) = 0.22, *p* = 0.048. Once again, no age group Endorsed the closer contestant above chance rates (see Table 3), and there was no correlation with age, *r*(79) = 0.07, *p* = 0.528. Additional correlations are reported in the Supplemental Material. Therefore, despite attempts to make the task easier for younger children, we found that many children in the sample did not use their judgment of who made lesser errors to inform their Ask and Endorse choices.

**Table 3:** Means and tests against chance for the selective social learning task, confidence task, and area task in experiments 3 and 4

	Experiment 3					Experiment 4				
	Mean (%)	SD	*t*	*p*	*d*	Mean (%)	SD	*t*	*p*	*d*
Winner										
4	51.19	23.02	0.24	0.815	0.05					
5	66.25	24.70	2.94	0.008	0.66	62.22	29.01	2.83	0.007	0.42
6	63.75	30.86	1.99	0.061	0.45	79.55	26.56	7.38	< 0.001	1.11
7	78.75	20.32	6.33	< 0.001	1.41	94.19	14.26	20.31	< 0.001	3.10
Ask										
4	54.76	24.52	0.89	0.384	0.19					
5	58.75	27.24	1.44	0.167	0.32	54.44	29.33	1.02	0.315	0.15
6	60.00	32.85	1.36	0.189	0.30	77.27	25.75	7.02	< 0.001	1.06
7	70.00	25.13	3.56	0.002	0.80	88.95	18.34	13.93	< 0.001	2.12
Endorse										
4	47.62	24.88	-0.44	0.666	0.10					
5	47.50	25.52	-0.44	0.666	0.10	50.00	26.11	0.00	1.00	0.00
6	55.00	27.63	0.81	0.428	0.18	64.77	31.12	3.15	0.003	0.47
7	51.25	28.65	0.20	0.847	0.04	66.86	28.72	3.85	< 0.001	0.59
Confidence										
4	59.76	8.29	5.40	< 0.001	1.18					
5	63.50	12.99	4.65	< 0.001	1.04	68.89	14.00	9.05	< 0.001	1.35
6	78.50	13.19	9.66	< 0.001	2.16	71.97	13.83	10.53	< 0.001	1.59
7	77.75	11.29	10.99	< 0.001	2.46	74.11	12.87	12.29	< 0.001	1.87
Area										
4	75.71	13.65	8.63	< 0.001	1.88					
5	82.62	5.29	27.60	< 0.001	6.17	83.56	9.51	23.66	< 0.001	3.53
6	84.88	7.88	19.78	< 0.001	4.42	86.70	8.69	28.02	< 0.001	4.22
7	84.25	3.98	38.47	< 0.001	8.60	86.05	8.13	29.06	< 0.001	4.43

#### Area task

Children at all ages accurately chose the larger shape well above chance levels (see Table 3), and accuracy increased with age, *r*(79) = 0.33, *p* < 0.001, replicating previous work (Odic, 2018).

#### Confidence task

Children at all ages chose the easier trial as their most certain more often than expected by chance (see Table 3), with a significant correlation with area discrimination, *r*(79) = 0.28, *p* = 0.011. Five children consistently chose the harder trial (three 4-year-olds and two 5-year-olds) and their data has been inverted. Additional analyses to replicate documented effects in this task are reported in the Supplemental Material.

#### Correlations of individual differences

Replicating Experiment 1 but *not* Experiment 2, children’s performance on the Confidence Task did not predict their Winner, *r*(78) = 0.14, *p* = 0.227, Ask, *r*(78) = 0.08, *p* = 0.499, or Endorse answers, *r*(78) = 0.12, *p* = 0.270, when controlling for age. Despite not seeing the predicted correlation, we felt that it could still be informative to examine how shared decision imprecision impacted performance. To do this, we conducted a hierarchical regression in two steps. In the first step, we included age and Area accuracy (our index of sensitivity to decision imprecision), and then added Confidence Task performance in the second step. The SDT/Simulation account predicts that Area accuracy should predict children’s social judgments in the first step, and that the Confidence measure should add no additional variability in the second step, given that the area and confidence measures are thought to tap the same decision imprecision. As shown in Table 4, however, we did not find evidence supporting this prediction. In step 1, there was no meaningful contribution of area accuracy, with age serving as the only significant predictor. In step 2, there was no meaningful contribution of confidence performance over and above age and area accuracy, but because area accuracy was not a meaningful predictor, this is further inconsistent with the SDT/Simulation account prediction. It appears, then, that an age-related change is responsible for the correlations between tasks rather than shared area representations.

**Table 4:** Hierarchical regressions in experiments 3 and 4

	Experiment 3								Experiment 4							
	Model Fit				Coefficients				Model Fit				Coefficients			
Model	*R*^2^	∆*R*^2^	*F*	*p*	Predictor	ß	*t*	*p*	*R*^2^	∆*R*^2^	*F*	*p*	Predictor	ß	*t*	*p*
DV: Winner																
Step 1	0.12	0.12	5.51	0.006	Age	0.35	3.10	0.003	0.19	0.19	15.14	< 0.001	Age	0.44	5.50	< 0.001
					Area Acc	0.01	0.10	0.923					Area Acc	-0.07	-0.87	0.386
Step 2	0.14	0.02	1.46	0.231	Age	0.26	1.99	0.050	0.19	0.00	0.03	0.864	Age	0.44	5.42	< 0.001
					Area Acc	-0.01	-0.05	0.963					Area Acc	-0.07	0.25	0.385
					Confidence	0.16	1.01	0.231					Confidence	-0.01	-0.17	0.864
DV: Ask																
Step 1	0.05	0.05	2.08	0.132	Age	0.20	1.71	0.091	0.20	0.20	16.08	< 0.001	Age	0.45	5.66	< 0.001
					Area Acc	0.06	0.47	0.637					Area Acc	-0.03	-0.35	0.731
Step 2	0.05	0.00	0.40	0.534	Age	0.15	1.11	0.269	0.20	0.00	0.01	0.920	Age	0.45	5.57	< 0.001
					Area Acc	0.05	0.40	0.694					Area Acc	-0.02	-0.35	0.730
					Confidence	0.09	0.62	0.534					Confidence	-0.01	-0.10	0.919
DV: Endorse																
Step 1	0.01	0.01	0.51	0.602	Age	0.10	0.86	0.393	0.03	0.03	2.00	0.139	Age	0.16	1.86	0.065
					Area Acc	-0.09	-0.79	0.433					Area Acc	0.05	0.53	0.599
Step 2	0.03	0.02	1.47	0.230	Age	0.01	0.09	0.931	0.03	0.00	0.15	0.704	Age	0.16	1.76	0.081
					Area Acc	-0.11	-0.93	0.356					Area Acc	0.05	0.54	0.593
					Confidence	0.17	1.21	0.230					Confidence	0.03	0.38	0.704

### Discussion

While we replicated some results of Experiments 1 and 2 in that children were selective in who to trust, and were sensitive to differences in confidence, we saw some major differences that cast doubt on the SDT/Simulation account. As in Experiment 1, there was no correlation between self and other judgments, and a hierarchical regression revealed that there was no influence of area accuracy on the three Selective Social Learning Task measures.

Once again, only 7-year-olds, the oldest children in our sample, showed above-chance selectivity to ask the closer informant for help though even 5-year-olds choose the closer informant as the winner. Their answers were above chance, though not significantly so, potentially meaning that we lacked sufficient power to detect these effects. While this alone cannot explain the pattern of correlations in the current experiment, we felt that it warranted a final study with a larger sample of children to provide a more definitive picture. A second possibility for the lack of correlations between self and other reasoning in Experiment 3 is the choice of a retrospective confidence task (as opposed to a prospective one in Experiments 1 and 2). Some have suggested that confidence signals derived before making a decision are distinct from those we make *after* a decision (Pouget et al., 2016), with confidence occurring before a decision relying more heavily on representational imprecision. To test both of these possibilities, we replicated Experiment 2, but assessed area accuracy through an additional task rather than embedding it into the confidence task as in Experiment 3.

## Experiment 4

### Methods

#### Participants

Using the observed correlation in Experiment 2 between children’s choice of Winner and performance on the Confidence task, we calculated that a sample size of 129 children would allow us to detect an effect with 0.90 power at α = 0.05. This sample size is also sufficient to detect an effect as small as *d* = 0.28 when comparing children’s selective social learning answers against chance. Rounding this sample up to counterbalance our stimuli, we tested 132 children (*M* = 6;5, range = 5;0—7;11, 82 girls) in the same manner as the other experiments. Three additional children were excluded for not completing the study in full, and none of the children in the sample had participated in the previous studies. As in Experiment 3, we asked parents to fill out the DVAP online within two weeks of participation. Forty-seven parents completed the assessment, and this data is reported in the Supplemental Material.

#### Materials and procedures

##### Selective social learning task

We used the same stimuli as in Experiment 3. Because our previous studies had not found that children at any age were selecting the closer informant on Endorse questions, we decided to modify the wording of this question slightly to clarify what we were asking. We asked children “Which girl’s shape would you guess looks the way it is supposed to look?,” which signaled that it was permissible to indicate the same girl as in previous responses (since it is ‘just a guess’), and highlighted the relation between the girls and the shapes in case children were only focusing on the features of the shapes.

##### Area task

Immediately after the Selective Social Learning task, children completed a 20-trial area discrimination task that served as a control for the similarities between the two key tasks. The task used the same type of stimuli as the confidence discrimination task (e.g., children chose the larger of two shapes), using ratios ranging from 1.05 to 3.3. Children were given pre-recorded feedback about the accuracy of their answer.

##### Confidence task

We used the prospective confidence task with only Area trials from Experiment 2, but without the warm-up trials as all children completed the area task first.

### Results

#### Selective social learning task

As shown in Table 3, children at all three ages identified the informant with lesser errors as the Winner more often than chance, with a significant increase with age, *r*(130) = 0.43, *p* < 0.001. Six- and 7-year-olds also Asked this informant for help, and in contrast with the previous studies also Endorsed her shape as being closer, while 5-year-olds did neither (see Table 3). Ask choices correlated with age, *r*(130) = 0.45, *p* < 0.001, and Endorse choices had trending correlation with age, *r*(130) = 0.17, *p* = 0.055. Additional correlations are reported in the Supplemental Material.

#### Area task

Children in all three age groups successfully identified the larger shape well above chance levels (see Table 3), and accuracy did not significantly increase with age, *r*(130) = 0.11, *p* = 0.213.

#### Confidence task

Children at all ages once again chose the easier question more than expected by chance (see Table 3). Eleven children consistently chose the harder trial (five 5-year-olds, two 6-year-olds, and four 7-year-olds) and their data has been inverted. There was no correlation with area performance, *r*(130) = -0.01, *p* = 0.939.

Age and area discrimination together did not significantly predict children’s confidence discrimination, *R*^2^ = 0.03, *F*(2, 129) = 2.06, *p* = 0.131, *R*^2^_Change_ from model with just age = 0.00, *F*(1, 129) = 0.09, *p* = 0.766, though the coefficients suggest that age was a more meaningful predictor than area, β_Age_ = 0.18, *t*(129) = 2.03, *p* = 0.044, β_Area_ = -0.03, *t*(129) = -0.3, *p* = 0.770, consistent with what we found in Experiment 3 and in contrast to the SDT account.

#### Correlations of individual differences

As in Experiments 1 and 3, there was no correlation between children’s confidence choices and their Winner, *r*(129) = -0.01, *p* = 0.908, Ask, *r*(129) = -0.01, *p* = 0.943, or Endorse choices *r*(129) = 0.03, *p* = 0.775, when controlling for age. We once again conducted a hierarchical regression with age and area accuracy in step 1 and added confidence performance in step 2, again finding no meaningful contribution of either area or confidence performance on the three social judgments.

### Discussion

We found that both tasks replicated the key patterns from past work, indicating that they were tapping into the target constructs. However, even when using a larger sample size powered to detect the observed effect size from Experiment 2, we did not replicate the correlation we previously found between children’s sensitivity to confidence and their selective social learning choices.

## Mega-analysis

Our failure to find a consistent correlation between self and other reliability judgements in three out of four experiments we ran suggests that – contrary to our initial predictions – the mechanisms supporting self vs. other judgements are distinct, or at least not following the theorized mechanistic link between SDT and Simulation Theory. At the same time, however, this conclusion relies on a null result. To quantify the strength of this null finding, we perform a mega-analysis, combining the results from the four experiments for maximal power.

To help clarify our findings, and particularly to determine whether we should interpret non-significance as evidence in favor of *no* effect, we computed Bayes Factors (BF). Bayes Factors provide the relative weight of the evidence for the null vs. the alternative hypotheses, and can therefore provide a measure of graded strength for the null hypothesis (Wagenmakers et al., 2018). If neither the null nor alternative hypotheses are supported, the model will output a BF_10_ of 1. If there is support for the alternative hypothesis, values will increase towards positive infinity, and if there is support for the null hypothesis, values will decrease towards 0. All Bayesian analyses were conducted in JASP with default priors.

### Selective social learning task

Combining the data from all four studies (*N* = 374), we find that children aged 5 and up gave Winner and Ask judgments consistent with our predictions and previous work, and children aged 6 and 7 gave consistent Endorse judgments (see Table 5). Thus, largely replicating the

**Table 5:** Means and tests against chance across all experiments

Age	Mean (%)	SD	*t*	*p*	*d*	BF_10_
Winner						
4	49.18	24.14	-0.27	0.792	0.03	0.15
5	62.38	62.38	4.80	< 0.001	0.46	2841.68
6	77.43	28.12	9.90	< 0.001	0.98	4.46 * 10^13^
7	87.86	20.07	19.15	< 0.001	1.89	1.77 * 10^32^
Ask						
4	51.23	23.46	0.41	0.684	0.05	0.15
5	58.88	27.53	3.34	0.001	0.32	18.75
6	68.20	31.15	5.93	< 0.001	0.58	280,741.29
7	80.34	24.91	12.36	< 0.001	1.22	8.42 * 10^18^
Endorse						
4	46.31	24.93	-1.16	0.252	0.15	0.26
5	50.23	27.36	0.09	0.930	0.01	0.11
6	58.25	29.58	2.83	0.006	0.28	4.70
7	58.73	30.60	2.60	0.011	0.29	8.74
Confidence						
4	62.65	11.93	8.28	< 0.001	1.06	5.61 * 10^8^
5	67.63	13.36	13.66	< 0.001	1.32	9.48 * 10^21^
6	71.68	14.17	15.53	< 0.001	1.53	2.66 * 10^25^
7	74.45	13.24	18.74	< 0.001	1.85	3.25 * 10^31^

findings of Einav and Robinson (2010), we found that children were sensitive to the relative degree of error, though we did not find evidence that 4-year-olds identified or strategically trusted relatively more accurate informants, with evidence moderately in favor of the null hypothesis.

### Correlations of Individual Differences

**Fig. 2:**
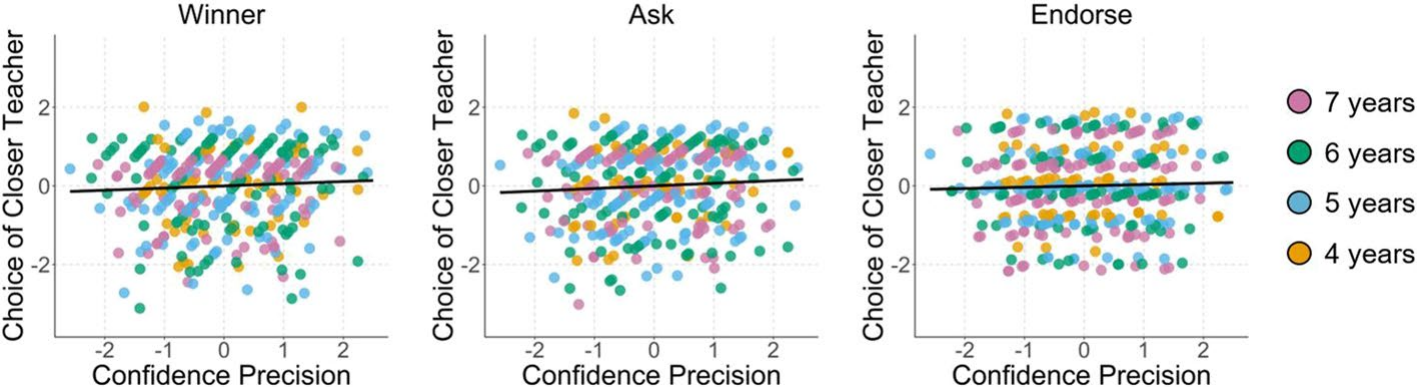
Correlations between confidence and social judgments controlling for age. Data plotted are standardized residuals

Combining the results from the four studies, there were no correlations between confidence sensitivity and Winner, *r*(371) = 0.06, *p* = 0.289, BF_10_ = 0.18, Ask, *r*(371) = 0.07, *p* = 0.202, BF_10_ = 0.28, or Endorse choices, *r*(371) = 0.03, *p* = 0.517, BF_10_ = 0.20, when controlling for age (see Fig. 2). Importantly, the Bayes Factors were less than 1/3 for including confidence performance as a predictor of the three Selective Social Learning variables, which can be interpreted as moderate evidence for the null hypothesis (i.e., that confidence sensitivity does *not* predict social judgments). By inverting the Bayes Factors, this null hypothesis is 5.71 times more likely than the hypothesis that confidence is a meaningful predictor for Winner judgments, 3.61 times more likely for Ask judgments, and 5.09 times more likely for Endorse judgments.

## General discussion

We set out to test an account linking reasoning about one’s own accuracy and others’ accuracy through SDT and Simulation Theory. This account hypothesized that confidence judgments are entirely calculated from decision imprecision, and that social reasoning is accomplished by simulating the confidence judgments of another. In four studies, we presented children with a social learning task and a confidence reasoning task that reduced shared mental state language and response biases, investigating whether imprecision in shared area representations alone led to correlations between the tasks. However, in three of the four studies, we found no evidence of a correlation between the self and other tasks, and a mega-analysis of the four studies revealed support in favor of *no* correlation. At the same time, both the Selective Social Learning and Confidence Tasks demonstrated sensible patterns replicating past work, indicating that the measures were tapping into the target constructs. Therefore, even under the most generous interpretation of our findings, in which the significant correlation in Experiment 2 represents the true correlation, there is a much weaker correlation than would be expected given the SDT/Simulation account hypotheses. Our interpretation, then, is that reasoning about the likelihood of one’s own accuracy and the likelihood of another’s accuracy are *not* computed by a single process rooted in representational variability.

Although our findings are not what we expected given previously reported correlations between self and other tasks, they are consistent with other recent evidence against an SDT account of confidence judgments. For example, using the same relative confidence task in 6–9-year-olds, Baer et al. (2018) reported correlations between confidence tasks that use independent representations, which should be uncorrelated according to pure SDT principles (and see Baer & Odic, 2020a). In the Supplemental Material, we partially replicate this finding using data from Experiment 1: area and number confidence judgments were correlated, but neither correlated with emotion confidence. Similarly, Maniscalco and Lau (2012) found that decision imprecision in adults did not perfectly predict confidence ability, again counter to the SDT prediction (see Baer & Odic, 2019 for similar developmental evidence). We replicate this finding in the Supplemental Material using the Confidence Tasks in Experiments 3 and 4, demonstrating that there is age-linked improvement in confidence reasoning beyond what can be explained by change in representational imprecision. Taken together, it seems increasingly unlikely that representational imprecision alone accounts for metacognitive confidence judgments, against the core SDT predictions.

We will note that the SDT account is still being tested with modifications that we do not account for in the current study. As one example, some SDT theories accommodate a mismatch between confidence and accuracy by inferring that there is additional variability in how well a participant can interpret the imprecision of their decision (e.g., Mamassian, 2020; Maniscalco & Lau, 2012; Rahnev et al., 2011). That is, under this view we should never expect representational imprecision to perfectly explain confidence judgments because there is a secondary process involved (e.g., akin to a statistician who calculates the standard error, but who sometimes presses the wrong number on the calculator or rounds off extra digits). As another example, some SDT theories posit a ‘winner-takes-all’ computation of confidence, whereby a participant computes confidence not as the difference or ratio between the decision variability, but rather focuses only on the strength of evidence for the chosen answer (e.g., Miyoshi & Lau, 2020; Zawadzka et al., 2017; Zylberberg et al., 2012). In our study, this could involve children tracking the overall statistics about ‘average’ shape size and inferring the most confidence for the largest shapes, regardless of their size relative to the other shape in the pair. As our study was not designed to test these versions of SDT theories, we cannot say for certain that SDT accounts in general cannot explain confidence judgments or judgments of other’s accuracy, only that the particular SDT account tested here does not.^4^

However, the lack of correlation alone does not conclusively rule out Simulation Theory as an explanation for reasoning about social judgments. For instance, while we designed our tasks to avoid certain response biases like overconfidence, there are still many potential biases that children could carry that operate in one task but not the other and could therefore serve to mask any underlying correlation. Children could have maximized their success in the Confidence Task as we intended, but instead maximized fairness over success in the Selective Social Learning Task by alternating between informants rather than consistently choosing the more accurate one (e.g., Shaw & Olson, 2012). This pattern would result in accurate measurement of the Confidence Task, but near chance-like performance on the Selective Social Learning Task. In other words, children could still have *understood* which informant was more accurate, but their performance might not have reflected this reasoning. We similarly know that there was some variability in children’s motivation in the Confidence Task from the presence of children who consistently chose the harder option, though they may have responded strategically in the Selective Social Learning Task as we anticipated. Though these patterns were likely uncommon given the above-chance performance on both tasks, these potential inconsistent motivations could have weakened any existing correlations. It is thus possible that children simulated their own reasoning when making judgments of others, but the resulting correlation was masked by conflicting motivational goals. Such response strategies would necessarily have to be much stronger than any individual differences in theorized shared mechanisms but are nonetheless plausible.

### Alternative theoretical accounts

One account that could accommodate our findings through a single common process comes from Bayesian accounts of cognition (Meyniel et al., 2015; Pouget et al., 2016). In most Bayesian theories, children’s decisions are based on weighted evidence, rationally combining prior expectations and available evidence from multiple sources (see Gopnik & Bonawitz, 2015). For this to work, all information must be available in a common unit: in this case, the probability of accuracy. Information like decision imprecision easily complies – more precision tends to indicate a higher probability of accuracy – but so could many other cues, such as one’s prior history of success or failure on the task, momentary distraction, mind wandering, and so on. For example, if by chance a teacher always smiles when telling the truth, then smiling could be construed as signalling accuracy. But notice that these same cues in a different context may sometimes signal errors: a teacher smiling during the *child’s* statement could reflect an attempt to encourage the child to work through an incorrect answer, or an answer with very high precision (that feels *too* easy) could hint that the question was misinterpreted. Cues are therefore not inherently diagnostic but must be interpreted by the child as meaningful for their decision.

Critically, a Bayesian account allows the child to determine how heavily each cue should be weighed for a given decision. If a child is familiar with ‘trick questions’ that feel easy but are actually difficult, then that child might not treat precision and the accompanying feelings of ease as a valid cue that they should be confident. In the same way, the cues that children deem relevant for their own decisions and for others’ decisions need not be the same. For instance, bodily cues like posture, facial expression, and reaction time may more heavily inform judgments of another’s accuracy than one’s own, while representational imprecision and feelings of ease may factor more heavily into judgments of one’s own accuracy (see Vuillaume et al., 2020). We might therefore not expect a correlation between self and other judgments if children weighed cues differently for the two judgment types, even with a single common Bayesian mechanism. Put differently: our results are consistent with self and other judgements relying on the same general process, but on distinct *information* (see also Thomas & Jacoby, 2013; Tullis, 2018).

A different account that advocates for a single underlying process of self and other reasoning is the *mindreading-first* account. Essentially the inverse of Simulation Theory, the mindreading-first account argues that all metacognitive reasoning is accomplished by turning one’s mindreading abilities inward (e.g., attending to one’s own behavioral cues as though observing the self; Carruthers, 2009; Gopnik, 1993). As with Simulation Theory, the lack of correlation between self and other tasks in the current work is inconsistent with this account. However, due to its focus on behavioral cues, the mindreading-first account presents one additional explanation for why we did not detect a correlation. In the Confidence Task, children could have attended to many cues including reaction time or states of anxiety (Carruthers, 2009; Koriat & Ackerman, 2010a; Paulus et al., 2014). However, in the Selective Social Learning Task, children did not see videos or live performances, and so did not have these or many other common behavioral cues that are known to impact children’s assessment of accuracy (Birch et al., 2010; Paulus et al., 2014). This imbalance in cues could in part explain why only children aged 5 and older performed above chance on the Selective Social Learning task, while even 4-year-olds succeeded at the Confidence Task where more cues were available. Future work would need to find ways of equating the kinds of cues that children attend to for themselves vs. for others in order to establish such a correlation.

As a final theoretical explanation, these findings are also consistent with theories suggesting full independence of self and other reasoning (e.g., Nichols & Stich, 2003). Though several studies have documented correlations (e.g., Gopnik & Astington, 1988; Kuzyk et al., 2020; Lockl & Schneider, 2007), they did not control for common features that may have induced correlations. Our experimental design eliminated two such features: common response biases (e.g., to seek as much information as possible) and common language (e.g., using mental state terms like “know”). Consistently, there was no correlation between self and other reasoning in young children in one study where these features were unaligned (Bernard et al., 2015). There, children were asked to opt out of an answer when uncertain (a metacognitive measure without the use of mental state terms and with reward-maximizing) and completed several classic theory of mind tasks (mindreading tasks with mental state terms but no personal investment). Together with the current work, these findings suggest that previously reported correlations could reflect other common features such as response biases or similar demands on mentalistic language, masking the potential independence of self and other reasoning.

Briefly, we will note that correlations driven by response biases or common language may still provide meaningful information about how children *use* self and other judgments. For example, one recent proposal is that response biases are a critical part of how learning occurs, potentially even more than the ability to distinguish between close estimates of accuracy that we isolated here (Baer & Odic, 2020b). That is, learning-relevant behaviors like choosing to seek help or discounting misinformation from others may depend much more heavily on how a child *interprets* their state of confidence (or evaluates whether another person is accurate *enough* to trust). Though our findings could suggest independence in how those signals of self and other accuracy are generated, common response biases gesture towards a single process responsible for *interpreting* those signals. The presence of common linguistic markers (“know,” “sure”) further supports this idea.

Our findings are also relevant to a recent proposal about the emergence of metacognitive ability during childhood which specifically relies on social learning (Heyes et al., 2020). The Cultural Origins hypothesis argues that children learn to reason metacognitively by watching others model good metacognition or by having a teacher guide them, rather than metacognition emerging either innately through genetic programming or through non-social experience. A critical prediction of this account is that children with good social skills (and in particular, good social learning skills that help them select the best teachers to learn from) should then show the best metacognitive skills (see Heyes et al., 2020, p. 358). Our findings are inconsistent with this account: children with good selective social learning performance (i.e., those children who selected the best teachers) did not have the best metacognitive skills.

### Replication of sensitivity to another’s error magnitude

These results also provide an important replication of the Selective Social Learning Task. Here, we asked children to reason about the relative accuracy of two informants by comparing the relative sizes of objects, rather than numerical estimates or categorical labels as used by Einav and Robinson (2010). Five-year-olds not only noticed the difference in sizes and attributed this to superior ability (by choosing the closer informant as the ‘Winner’), they also used this attribution to strategically ask for help (“Ask” questions) and by age 6 to reason about ambiguous cases (“Endorse” questions). We can therefore echo Einav and Robinson’s (2010) conclusion that children are sensitive to the magnitude of an informant’s error and do not reason about accuracy only in binary terms.

We do, however, see some differences in our pattern of results compared to the original findings of Einav and Robinson (2010). Most notably, 4-year-olds did not attribute any differences in the magnitude of error to the informants’ ability, nor did they selectively Ask or Endorse the closer informant. This is not likely due to difficulty reasoning about area judgments relative to number judgments, as there is ample evidence that reasoning about area is well-developed by this age (and certainly for the ratios used in this task), while numerical reasoning continues to develop into later childhood (Odic, 2018). Instead, we suspect that there are three likely (and not mutually-exclusive) explanations. First, our task could have been less engaging for children because of the use of pictures rather than videos or live demonstrations, and so these youngest children could have been unmotivated to respond strategically. Second, children were not given a number line to record the answers of each informant, as they were in the Einav & Robinson study, which could mean that 4-year-olds understand the general principle that ‘closer is better’, but do not spontaneously track the magnitude of error without help. Third, it could also be that 4-year-olds do not possess a ‘closer is better’ rule at all, as 4 and 5-year-olds were considered as a single age group in Einav & Robinson’s study, so older children could have driven their effect.

We also found that children in Experiments 1–3 did not strategically Endorse the closer informant, even though they chose her more often than chance in both Winner and Ask questions. This changed in Experiment 4 with a seemingly small difference in the question wording: from “Which one do you think is more like the [target shape]?” to “Which girl’s shape would you guess looks the way it is supposed to look?” This change introduces two possible explanations for differences across studies. One is that by drawing attention to the informants as the owners of the shapes, children may have been more likely to think about the informants and their abilities than without this cue. The second is that by using language that acknowledges the ambiguity and imperfection of the situation (“would you guess” and “supposed to look”), children may have felt more comfortable repeating their choice of informant (e.g., Bonawitz et al., 2020) or overriding a desire to be fair to both informants due to plausible deniability (e.g., Shaw et al., 2014). Future work testing each of these possibilities could be useful not only in understanding children’s behavior on selective learning tasks, but more generally in understanding how children balance epistemic and social goals (Jaswal & Kondrad, 2016; Landrum et al., 2015).

### Replication of relative confidence reasoning

We also replicated and extended the findings of Baer and Odic (2019; Baer et al., 2018) in the relative confidence task. As reported in their studies, children responded to their confidence in area discriminations by selecting the easier question (Experiments 1, 2, and 4) or the more accurate answer (Experiment 3). We further replicated their findings of age-related improvement, and in the Supplemental Material show that this is not due to developing area reasoning. Extending this work, we saw that even 4-year-olds were significantly above chance in their confidence reasoning in all four studies. This is currently the youngest age group reported to compare their confidence between two questions, supporting the prediction made by Baer and Odic (2019) that children under age 5 may show sensitivity to confidence if given large enough contrasts in difficulty or an easier task (as the area task is relative to the number task, see Odic, 2018).

### Conclusion

Overall, we did not find evidence to support the SDT/Simulation account, and in fact found ample evidence *against* the SDT predictions. Our primary conclusion is therefore that children do not reason about both self and others through a single, representational-imprecision-driven process, as predicted by SDT. These results also point towards no correlation between self and other reasoning, though as outlined, alternative non-SDT or non-simulation-based theoretical accounts could still accommodate this evidence with a single process, which warrants further investigation. However, these experiments *do* support claims that children can reason strategically about both their own and another’s reliability, demonstrating flexibility in their learning mechanisms through tools that help navigate truth from fiction.

## Supplementary Information

Below is the link to the electronic supplementary material.

Supplementary file1 (DOCX 26.2 KB)

## Data Availability

Stimuli, data, and associated extraction and analysis scripts (in R) are available on OSF at https://osf.io/dtzpq/.

## Footnotes

1 Note that because our main task uses a 2-alternative forced choice format (choosing which shape is larger), we are specifically referring to theories of SDT that propose confidence is a relative computation of decision imprecision. Other variations of SDT theories are briefly discussed in the General Discussion.

2 Baer et al. (2018) did find correlations between these three tasks in children aged 6–9, slightly older than we tested here. However, other findings using these same dimensions (e.g., Vo et al., 2014) and using other dimensions (Bellon et al., 2020; Geurten et al., 2018) show no correlations between metacognitive dimensions under age 8.

3 Note that these data and those from Experiment 2 are included as unpublished data in this meta-analysis.

4 Because we ensured that high confidence trials did not always feature the largest shape, we had trials in which size was inconsistent with expected confidence (e.g., the largest shape was part of a difficult trial, about 40% of trials). Inconsistent with the winner-takes-all account, children still chose the trial with a higher ratio rather than the one with the largest shape more than chance (p’s < .003) which may mean that the relative confidence format discourages this heuristic.
