# Supplementary material for: Are children’s judgments of another’s accuracy linked to their metacognitive confidence judgments?
Source: Metacogn Learn. 2021 Mar 27;16(2):485–516. doi: 10.1007/s11409-021-09263-x (PMC8550463; doi:10.1007/s11409-021-09263-x)
Supplement: Supplementary file 1 — Supplementary file1 (DOCX 26.2 KB) [file 11409_2021_9263_MOESM1_ESM.docx]

# Supplemental Material

## Correlations Between Selective Social Learning Measures

To explore whether the three responses (Winner, Ask, and Endorse) tapped into the same social reasoning process, we examined their intercorrelations, that is, whether children would consistently choose the same contestant across the three trials (e.g., Einav & Robinson, 2010; Koenig & Harris, 2005). All correlations control for age.

Experiment 1. Consistent with previously published findings, children’s Ask choices were correlated with their choice of Winner when controlling for age, *r*(77) = .34, *p* = .002. Children’s Endorse choices were not correlated with their choice of Winner, *r*(77) = .03, *p* = .760, and or with their Ask choices, *r*(77) = .18, *p* = .114.

Experiment 2. As in Experiment 1, children’s choice of Winner was related to who they Asked for help, *r*(78) = .58, *p* < .001, but not with who they Endorsed, *r*(78) = .11, *p* = .339. There was a trending correlation between Ask and Endorse choices that did not reach significance, *r*(78) = .21, *p* = .067.

Experiment 3. Children’s choice of Winner was related to who they Asked for help, *r*(78) = .43, *p* < .001, and Ask judgments correlated with Endorse judgments, *r*(78) = .28, *p* = .013, but Endorse responses did not significantly relate to choice of Winner, *r*(78) = .19, *p* = .085.

Experiment 4. Children’s choice of Winner was correlated with who they Asked for help, *r*(129) = .55, *p* < .001, and who they Endorsed, *r*(129) = .39, *p* < .001, and Ask and Endorse responses were also correlated, *r*(129) = .40, *p* < .001.

## Additional Confidence Task Analyses

Experiment 1. We first examined whether children’s performance depended on the metaratio – the difference in difficulties between the two presented trials. In particular, we were interested to see if children were more likely to choose the easier option when the ratio was large (meaning that there was a greater difference in difficulty between the two options), as reported in other studies using this measure (Baer et al., 2018; Baer & Odic, 2019). We conducted a repeated-measures ANOVA on the three metaratios (1.33, 2.0, and 3.0) with Age Group (4, 5, 6, or 7) as a between-subjects factor for each of the three dimensions. For Area, we found that children were more likely to choose the easier question as they got older, *F*(3, 76) = 3.89, *p* = .012, η_p_^2^ = 0.13, but were not more likely to choose the easier question as the difference in difficulties increased, *F*(2, 152) = 1.37, *p* = .257, η_p_^2^ = .02. There was no interaction between the two, *F*(6, 152) = 1.48, *p* = .188, η_p_^2^ = .06. For Number, children were more likely to choose the easier question as the difference in difficulties increased, *F*(2, 152) = 10.35, *p* < .001, η_p_^2^ = .12, but without a developmental effect, *F*(3, 76) = 1.02, *p* = .391, η_p_^2^ = .04, and no interaction, *F*(6, 152) = 0.14, *p* = .991, η_p_^2^ = .00. For Emotion, children were more likely to choose the easier question as the difference in difficulties increased, *F*(2, 152) = 12.50, *p* < .001, η_p_^2^ = .14, but without a developmental effect, *F*(3, 76) = 0.62, *p* = .602, η_p_^2^ = .02, and no interaction, *F*(6, 152) = 0.57, *p* = .757, η_p_^2^ = .02.

As an additional test of the SDT account, we next examined whether children’s metacognitive tendency to pick the easier question correlated between the three types of trials. Under the SDT framework, confidence judgments are derived solely from representational imprecision – area representations for area judgments and number representations for number judgments. Importantly, past work suggests that these representations are independent from one another: accuracy at choosing the larger shape does not correlate with accuracy at choosing the larger number of dots or the happier expression, and these abilities follow different developmental trajectories (Baer et al., 2018; Odic, 2018; Vo et al., 2014). The SDT account therefore predicts that confidence judgments using these representations would similarly be independent (Baer et al., 2018; Vo et al., 2014). Consistently, we found no correlation between Emotion and Area confidence choices when controlling for age, *r*(77) = .03, *p* = .776, and no significant correlation between Emotion and Number confidence choices, *r*(77) = .18, *p* = .119, suggesting that Emotion confidence judgments were independent of Number and Area confidence. However, we found that Number and Area confidence choices were correlated, *r*(77) = .34, *p* = .002, against the predictions of the SDT account but consistent with recent work for children older than 6 (Baer et al., 2018). We report correlations of the Selective Social Learning Task with all three confidence tasks in the main text, but these data suggest that the Emotion choices are more likely to reflect the representational independence required to test the SDT/simulation account.

Experiment 2. In a repeated-measures ANOVA on the three metaratios (1.33, 2.0, and 3.0) with Age Group (4, 5, 6, or 7) as a between-subjects factor, we did not find any effect of metaratio, F(2, 154) = 2.49, p = .087, η_p_^2^ = .03, age group, F(3, 77) = 0.37, p = .775, η_p_^2^ = .01, or their interaction, F(6, 154) = 0.66, p = .681, η_p_^2^ = .02.

Experiment 3. In an ANOVA on the 4 metaratios (1.1, 1.33, 2.0, and 3.0) with age group as a between-subjects factor, we found that children were more likely to choose the easier question as they got older, F(3, 77) = 14.16, p < .001, η_p_^2^ = .36, and were more likely to choose the easier question as the difference in difficulties increased, F(3, 231) = 16.65, p < .001, η_p_^2^ = .18. There was no interaction between the two, F(9, 231) = 0.45, p = .907, η_p_^2^ = .02.

Because we had a measure of both area accuracy and confidence choices, we also looked for two effects shown in number confidence decisions. First, we examined whether children’s confidence judgments reflected their actual accuracy: was area discrimination accuracy higher on trials children chose to keep (e.g., high confidence) compared to discarded trials (low confidence)? Replicating past findings, we found that accuracy on high confidence trials (*M* = 86.23, *SD* = 12.59) was higher than accuracy on low confidence trials (*M* = 77.35, *SD* = 10.99), *t*(80) = 5.44, *p* < .001, *d* = 0.75 (Baer & Odic, 2019).^[[1]](#footnote-1)^ Second, recent work has argued against the SDT account by demonstrating that age-related change in confidence choices was not solely accounted for by representational imprecision, and therefore that the SDT account of metacognitive confidence is incomplete (Baer & Odic, 2019). To examine this claim in the current study, we conducted a hierarchical regression predicting whether children would choose the easier trial (representing good metacognitive reasoning). Of interest was whether there was remaining age-related variability after including area performance in the model. Consistent with these past findings, adding area performance to a regression including age did not explain any additional variability in children’s choice of the easier trial, *R*^2^ = .34, *F*(2, 78) = 19.96, *p* < .001, *R*^2^_Change_ = .01, *F*(1, 78) = 1.10, *p* = .297, β_Age_ = .54, *t*(78) = 5.53, *p* < .001, β_Area_ = .10, *t*(78) = 1.05, *p* = .300. Together, these findings suggest that children’s confidence choices were appropriately predictive of their accuracy, and that individual differences in area performance were insufficient to completely explain confidence choices.

Experiment 4. Children were more likely to choose the easier question on larger metaratios, *F*(2, 258) = 6.90, *p* = .001, η_p_^2^ = .05, but there was no improvement with age, *F*(2, 129) = 1.26, *p* = .286, η_p_^2^ = .02, and no interaction, *F*(4, 258) = 0.18, *p* = .950, η_p_^2^ = .00.

Age and area discrimination together did not significantly predict children’s confidence discrimination, *R*^2^ = .03, *F*(2, 129) = 2.06, *p* = .131, *R*^2^_Change_ from model with just age = .00, *F*(1, 129) = 0.09, *p* = .766, though the coefficients suggest that age was a more meaningful predictor than area, consistent with what we found in Experiment 3, β_Age_ = .18, *t*(129) = 2.03, *p* = .044, β_Area_ = -.03, *t*(129) = -0.3, *p* = .770.

## Vocabulary Assessment

In Experiments 3 and 4, we collected additional data about children’s vocabulary through a parent report (the Developmental Vocabulary Assessment for Parents, or DVAP; Libertus et al., 2015), but only obtained data for a third of our sample. With these data combined across the studies (*N* = 72, *M* = 115.68, *SD* = 33.67), we found that DVAP scores increased with age, *r*(70) = .30, *p* = .011, as expected. When controlling for age, DVAP scores correlated with children’s choice of Winner, *r*(69) = .40, *p* < .001, and who they Asked for help, *r*(69) = .25, *p* = .032, but not who they Endorsed, *r*(69) = .08, *p* = .502, or their performance on the confidence task, *r*(69) = .06, *p* = .648.

**References**

Baer, C., Gill, I. K., & Odic, D. (2018). A domain-general sense of confidence in children. *Open Mind: Discoveries in Cognitive Science*, *2*, 86–96. https://doi.org/10.1162/opmi_a_00020

Baer, C., & Odic, D. (2019). Certainty in numerical judgments develops independently of the Approximate Number System. *Cognitive Development*, *52*, 100817. https://doi.org/10.1016/j.cogdev.2019.100817

Libertus, M. E., Odic, D., Feigenson, L., & Halberda, J. (2015). A Developmental Vocabulary Assessment for Parents (DVAP): Validating parental report of vocabulary size in 2- to 7-year-old children. *Journal of Cognition and Development*, *16*, 442–454. https://doi.org/10.1080/15248372.2013.835312

Odic, D. (2018). Children’s intuitive sense of number develops independently of their perception of area, density, length, and time. *Developmental Science*, *21*, e12533. https://doi.org/10.1111/desc.12533

Vo, V. A., Li, R., Kornell, N., Pouget, A., & Cantlon, J. F. (2014). Young children bet on their numerical skills metacognition in the numerical domain. *Psychological Science*, *25*, 1712–1721. https://doi.org/10.1177/0956797614538458

1. Note that for this analysis, we did not invert any data based on the psychophysical model. [↑](#footnote-ref-1)
